# Smart Lipid–Polysaccharide Nanoparticles for Targeted Delivery of Doxorubicin to Breast Cancer Cells

**DOI:** 10.3390/ijms23042386

**Published:** 2022-02-21

**Authors:** Manuela Curcio, Matteo Brindisi, Giuseppe Cirillo, Luca Frattaruolo, Antonella Leggio, Vittoria Rago, Fiore Pasquale Nicoletta, Anna Rita Cappello, Francesca Iemma

**Affiliations:** Department of Pharmacy, Health and Nutritional Sciences, University of Calabria, 87036 Rende, CS, Italy; matteo.brindisi@unical.it (M.B.); luca.frattaruolo@unical.it (L.F.); antonella.leggio@unical.it (A.L.); vittoria.rago@unical.it (V.R.); fiore.nicoletta@unical.it (F.P.N.); annarita.cappello@unical.it (A.R.C.); francesca.iemma@unical.it (F.I.)

**Keywords:** lipid–polymer nanoparticles, breast cancer, targeted drug delivery, stimuli-responsivity, CD44 receptors

## Abstract

In this study, actively-targeted (CD44-receptors) and dual stimuli (pH/redox)-responsive lipid–polymer nanoparticles were proposed as a delivery vehicle of doxorubicin hydrochloride in triple negative breast cancer cell lines. A phosphatidylcholine lipid film was hydrated with a solution of oxidized hyaluronic acid and doxorubicin, chosen as model drug, followed by a crosslinking reaction with cystamine hydrochloride. The obtained spherical nanoparticles (mean diameter of 30 nm) were found to be efficiently internalized in cancer cells by a receptor-mediated endocytosis process, and to modulate the drug release depending on the pH and redox potential of the surrounding medium. In vitro cytotoxicity assays demonstrated the safety and efficacy of the nanoparticles in enhancing the cytotoxic effect of the free anticancer drug, with the IC_50_ values being reduced by two and three times in MDA-MB-468 and MDA-MB-231, respectively. The combination of self-assembled phospholipid molecules with a polysaccharide counterpart acting as receptor ligand, and stimuli-responsive chemical moieties, was carried out on smart multifunctional nanoparticles able to actively target breast cancer cells and improve the in vitro anticancer activity of doxorubicin.

## 1. Introduction

Breast cancer is the malignant neoplasm with the highest incidence in women in developed countries [1]. Surgery, radiotherapy and chemotherapy are the common clinical approaches to eradicate and/or reduce the tumor mass, with the administration of anticancer drugs typically accompanied by severe side effects related to their poor selectivity, and by the insurgence of multidrug resistance [2], affecting the success of the treatment. Thus, in recent years, research activity in the pharmaceutical and biomedical fields has focused on the design of new formulations able to vectorize the antineoplastic agent to the tumor site, optimizing its pharmacokinetic profile and limiting the adverse side effects [3,4]. Actively targetable nanoparticles have been proposed as ideal tools for this purpose by virtue of their ability to efficiently accumulate in the tumor site [5], selectively interact with the cancer cells and promptly respond to the pathological signals from the surrounding environment, thereby modulating the drug release [6]. Typically, the targeting activity is conferred by either the functionalization with macromolecules/small compounds (i.e., transferrin, hyaluronic acid, folic acid), acting as ligands of membrane receptors overexpressed on cancer cells [6,7], or the insertion of chemical groups able to destabilize the nanoparticle structure exploiting the differences of environmental parameters (pH, temperature and redox state) between the intra- and extracellular spaces [8].

Among the drug carriers proposed for cancer therapy, nanoparticles deriving from the self-assembly of phospholipid materials, named liposomes, have attracted much attention due to their similarity with cell membranes, making them able to easily enter the cells and enhance the intracellular delivery and the retention time of the anticancer drug [9]. Nevertheless, they suffer from some drawbacks related to the poor site-specificity and lack of structural integrity [10,11]. In addition, when administered intravenously, liposomes show a short half-life due to phagocytosis by the components of the reticuloendothelial system [12]. Hydrophilic polymers, such as PEG [13] and polysaccharides [14], have been proposed as functionalizing agents to prolong the circulation time and confer “stealth” properties, while multi-targeted liposomes have been obtained by derivatization with suitable receptor ligands, as well as by insertion of chemical groups able to respond to pathological signals from the disease site [15]. Due to its ability to bind, the CD44 receptors have been shown to overexpress in many solid tumors, and the polysaccharide hyaluronic acid (HA) to be successfully employed as targeting elements of liposomal formulations by either covalent [16,17,18] or non-covalent [19,20] functionalization. Furthermore, the presence of the carboxylic moieties in the repeating units [21] makes the polysaccharide susceptible to chemical modification with stimuli-responsive specimens, enhancing its versatility as a targeting component [22,23,24].

In this study, the advantageous features of liposomes and HA were combined in a new phospholipid–polysaccharide nanoparticle architecture (PHYN) with active targeting properties and pH/redox-responsive activity. To the best of our knowledge, this is the first example of phospholipid–polysaccharide hybrid material endowed with all these valuable properties.

In detail, PHYN is composed of a phosphatidylcholine (PDC) bilayer and an oxidized HA (oxHA) crosslinked with disulfide bonds (cystamine hydrochloride—Cys) mainly confined in the outer surface of the bilayer structure. To test the suitability of the proposed system as a targeted drug delivery vehicle, nanoparticles were loaded with doxorubicin (DOX), a potent antineoplastic drug belonging to the anthracycline antibiotics group [25].

The goal of the study was to obtain targeted nanoparticles stable in the systemic circulation and to destabilize them in the intracellular compartment of tumor cells, triggering the drug release by virtue of both the acid-labile imine linkages between oxHA and Cys and the disulfide bridges of Cys moieties.

The obtained system was characterized in terms of physico–chemical features (size, shape and surface charge) and, after determining the cellular uptake efficiency, the influence of different pH and redox conditions over the drug release profile was assessed. Then, the antitumor performance was investigated in terms of cytotoxicity on MCF-10A (non-tumorigenic cells) and two triple negative breast cancer cell lines (MDA-MB-231 and MDA-MB-468). Finally, the ability of nanoparticle formulation to induce apoptosis in cancer cells was determined.

## 2. Results and Discussion

### 2.1. Synthesis and Characterization of Nanoparticles

**Figure 1:**
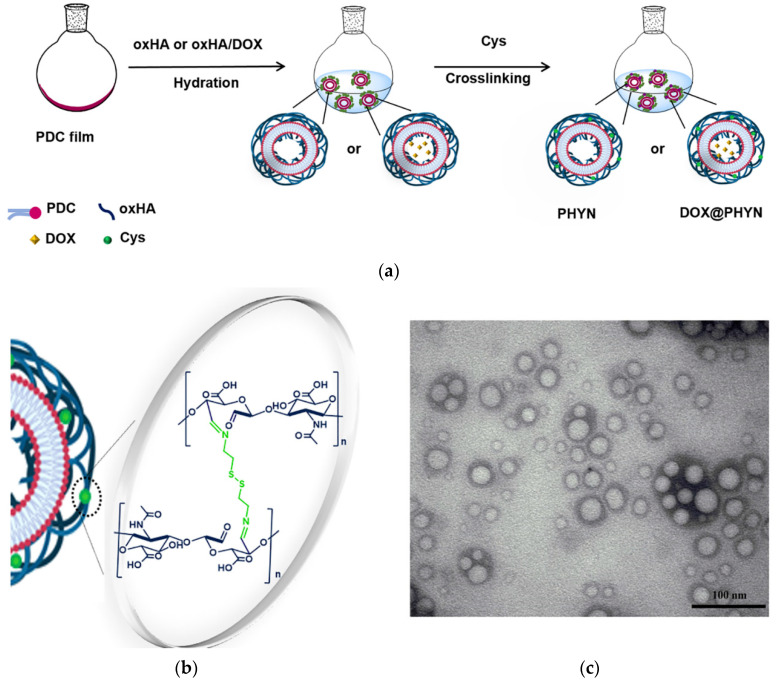
(**a**) Schematic representation of PHYN and DOX@PHYN preparation by modified thin film hydration method; (**b**) crosslinking reaction via Shiff-base formation between oxHA and Cys; (**c**) TEM image of negatively stained PHYN.

The combination of materials with different chemical and functional features represents a valuable strategy to obtain systems with superior properties and improved performances [26,27]. Here, redox-sensitive and CD44 receptor-targeted nanoparticles were fabricated by a modified thin film method employing PDC and oxHA as phospholipid and polymeric components, respectively, and Cys as disulfide bonds-containing specimen (Figure 1a).

**Figure 2:**
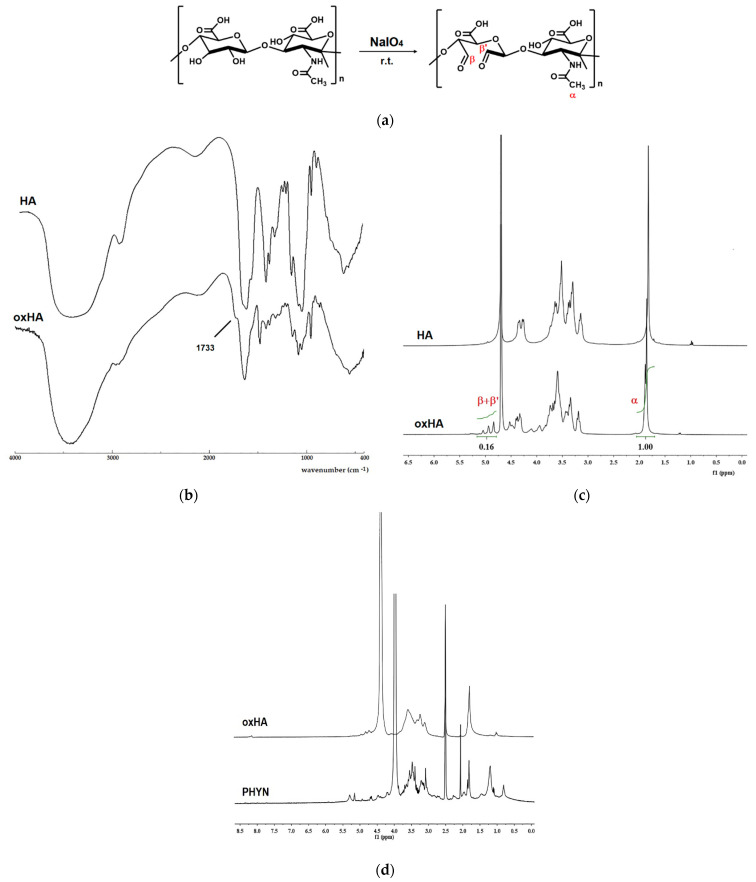
(**a**) Synthesis of oxHA; (**b**) FT-IR spectra of HA and oxHA; (**c**) ^1^H-NMR spectra of HA and oxHA in D_2_O; (**d**) ^1^H-NMR spectra of oxHA and PHYN nanoparticles in DMSO_d6_/D_2_O mixture.

Firstly, oxHA was prepared by reaction of HA with NaIO_4_, one the most useful oxidizing agent for polysaccharide molecules [28]. The reaction carried out to the oxidation of vicinal hydroxyl groups on C-2 and C-3 of the HA repetitive units to aldehydes, as well as the rupture of the C-C bonds (Figure 2a).

The presence of the aldehyde groups in oxHA was confirmed by the appearance of an absorption band at 1733 cm^−1^ in an FT-IR spectrum (Figure 2b), assigned to the stretching vibrations of aldehydic groups, as well as by the presence in the ^1^H-NMR spectrum (Figure 2c) of the signals at 4.80–5.10 ppm corresponding to the protons of aldehyde hydrates (HA-CH(OH)_2_) formed in D_2_O [29]. A degree of oxidation of about 25% was calculated by comparing the integral of aldehyde hydrates to that of the acetamide signal at 1.85 ppm.

Then, PHYN were obtained by hydrating a PDC thin film with oxHA solution in PBS (25% *w*/*w* PDC), followed by the addition of Cys to the colloidal dispersion, leading to the formation of acid-labile crosslinking points (Schiff-base groups) with the aldehyde moieties of oxHA (Figure 1b). An excess of Cys was employed in order to confer the highest redox responsivity to the nanoparticles, thus ensuring an efficient triggering of the drug release.

By this process, a homogenous spherical nanoparticle population, with a mean hydrodynamic diameter (35 ± 6 nm) and a PDI of 0.22 (as per dynamic light scattering (DLS) analysis), was obtained, as also confirmed by transmission electron micrography (TEM) investigations (Figure 1c). It can be hypothesized that they consist of a complex vesicle structure in which oxHA was organized in the aqueous compartments of the PDC bilayer, mainly on the outer surface. The measurement of the electric charge close to the particle surface confirmed this hypothesis, with a negative ζ-potential (−51 mV ± 2 mV at 25 °C) being attributed to the anionic character of oxHA on the nanoparticles surface, in accordance with literature data describing HA coated vesicular structures [30,31]. Moreover, since the magnitude of ζ-potential gives a prediction of the colloidal stability (colloidal systems with values >+30 mV and <−30 mV are electrically stabilized), such value was used to claim for stable nanoparticles [32]. The stability of PHYN formulation over time was evaluated by the measurement of zeta potential and the mean hydrodynamic stability for 15 days at 25 °C, finding that both the parameters remained almost unmodified (data not shown).

The composition of PHYN nanoparticles was then verified by ^1^H-NMR spectrum (Figure 2d), showing the resonances at about 0.81 ppm and in the region 1.01–1.58 ppm corresponding to the terminal methyl groups and methylene protons ((CH_2_)_n_) of the hydrocarbon chains of PDC moiety, respectively, as well as the signal at 1.82 ppm, assigned to the protons of the HA acetyl groups. The signals of protons related to HA disaccharide repeating units appeared at 3.01–3.80 ppm, while the signals at about 5.15 and 5.30 ppm corresponded to the methine hydrogen of glycerol backbone and to the olefinic protons of PDC, respectively [33,34].

The crosslinking degree was estimated after complete de-crosslinking of PHYN nanoparticles via reduction of the disulfide bonds within Cys moieties and measuring the SH amount by Ellman’s assay [35]. It is known that in the presence of an excess of 2-mercaptoethanol, each disulfide bond is reduced to two cysteamine residues linked to two *CHO* groups of oxHA by Schiff bases. Therefore, the crosslinking degree (*CD*) can be expressed as the amount (%) of conjugated to total (as per ^1^H-NMR analyses) aldehyde groups according to the following Equation (1):

(1) $$CD\left(\%\right)=\frac{mol_{SH}}{mol_{CHO}}\times 100$$

where *mol_SH_* and *mol_CHO_* were the amount (mol) of thiols and aldehyde groups in the sample, respectively.

In our conditions, a *CD* of 67% was obtained, indicating that more than half of available *CHO* groups within PHYN nanostructures were involved in the formation of the crosslinking points.

### 2.2. CD44-Mediated Cellular Uptake of Nanoparticles

PHYN nanoparticles were designed as DOX intracellular delivery vehicles targeting the tumor cells by means of HA moieties on an external surface [36]. DOX was loaded into the aqueous core of PHYN nanoparticles during the hydration step obtaining a post-dialysis EE of 85%. Before evaluating the release profile, the ability of the nanosystem to be internalized in cancer cells by the CD44-mediated process was investigated by exploiting the DOX intrinsic fluorescence properties.

**Figure 3:**
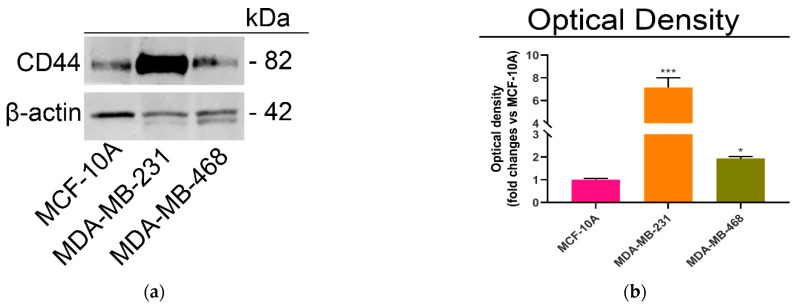
(**a**) Immunoblot analysis of CD44 expression levels in MCF-10A, MDA-MB-231 and MDA-MB-468, (**b**) Histograms representing the optical density. Values are expressed as means ± SD (*n* = 3) from three independent experiments. * *p* < 0.05; *** *p* < 0.001 vs. control.

At first, the expression levels of this receptor were determined by immunoblot analysis in two different and highly aggressive triple negative breast cancer lines, MDA-MB-231 (mesenchymal stem-like; basal B) and MDA-MB-468 (basal-like 1; basal A) and healthy MCF-10A breast cells (Figure 3a).

As expected, the immunoblot results showed the highest expression levels of CD44 in MDA-MB-231, followed by MDA-MB-468, and MCF-10A cells (Figure 3b).

**Figure 4:**
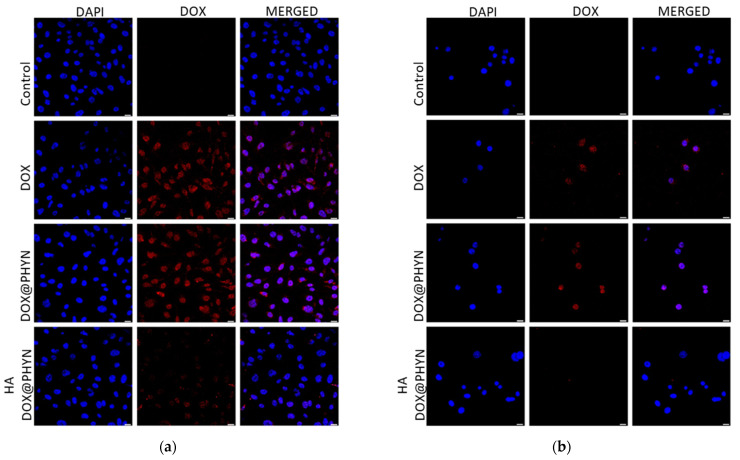

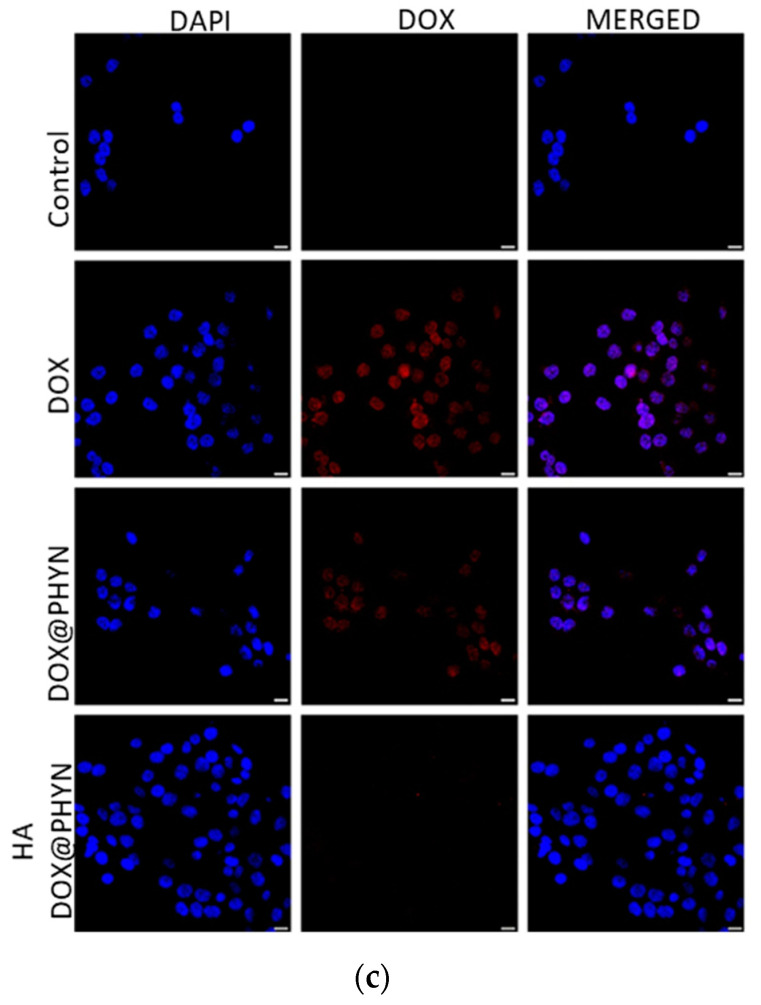
DOX@PHYN uptake depends on both CD44 expression and function. Confocal images of (**a**) MDA-MB-231, (**b**) MDA-MB-468 and (**c**) MCF-10A exposed to DOX or DOX@PHYN for 24 h. MDA-MB-231, MDA-MB-468 and MCF-10A were also exposed to hyaluronic acid, to block CD44 receptors, and DOX@PHYN (as indicated). Red fluorescence corresponds to doxorubicin fluorescence. DAPI was used to stain nuclei. Cells exposed to PHYN were used as the control. Pictures were obtained at 20×. Scale bar 25 µm.

Then, the selective targeting of DOX@PHYN to CD44 (+) (MDA-MB-231 and MDA-MB-468) or CD44 (−) (MCF-10A) cells was evaluated by confocal analysis (Figure 4).

Compared to the free form of the drug, DOX@PHYN nanoparticles showed enhanced ability to enter those cancer cells with the highest intensity of fluorescence signal recorded in MDA-MB-231 cells as a consequence of the highest CD44 levels. On the other hand, a constant or slightly reduced uptake of the nanoparticles vs. free DOX was observed in the MCF-10A cell line.

Moreover, to evaluate the efficiency of HA/CD44-mediated specific cellular uptake, further experiments were performed by preliminarily blocking CD44 receptors with free HA before incubation with DOX@PHYN nanoparticles. The results clearly proved that the pretreatment with HA was able to compete with the DOX@PHYN uptake, thus confirming the nanoparticles’ CD44-mediated internalization mechanism.

### 2.3. DOX Release Experiments

In addition to the specific interaction with CD44 receptors, the proposed nanoparticle systems were endowed with chemical groups able to respond to unique internal stimuli from the tumor tissue. Indeed, it is well known that the pH of tumor tissues is lower than that of both normal cells and blood (7.4) due to the high level of lactic acid generated by a deregulated glycolysis [37,38], and that the intracellular trafficking of pH-sensitive nanoparticles is associated with pH values of 4.5–6.5 into lysosomes [39,40]. On the other hand, the cytoplasmatic glutathione (GSH) concentration is 4–6 folds higher in cancer than in normal cells [41]. Thus, by endowing the nanoparticles with ionizable/acid-labile and reducible chemical groups, materials able to release the payload in response to the signals from the tumor microenvironment can be obtained.

**Figure 5:**
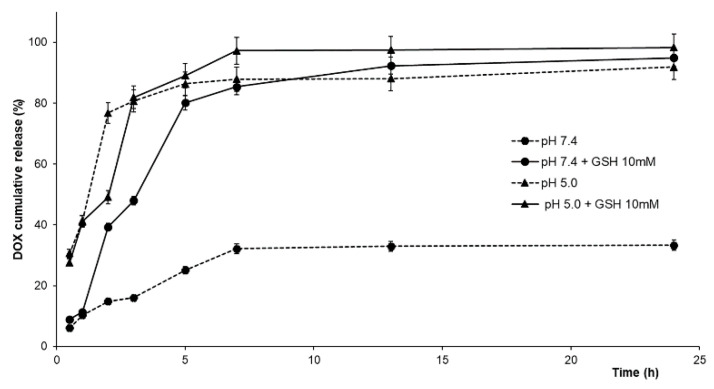
DOX release from DOX@PHYN at 37 °C in different pH and redox conditions.

In our experimental conditions, in order to simulate the extracellular and intracellular conditions, release experiments were performed in phosphate buffer (pH 7.4) and acetate buffer (pH 5.0) containing GSH at different concentrations (0 and 10.0 mM) [42,43] (Figure 5).

In physiological conditions, DOX@PHYN was able to deliver the encapsulated drug in a controlled manner, and a slow release profile was recorded, with DOX percentages not exceeding 33% after 24 h. The addition of GSH in the release medium led to a marked enhancement of the released DOX (40 and 85% at 2 and 24 h, respectively), as a consequence of the reduction of disulfide bridges in the nanoparticles structure. On the other hand, at pH 5.0, increased release percentages were recorded from the earliest experimental times (~30% and 80% after 30 min and 3 h, respectively), due to the breakage of the acid-labile oxHA-Cys imine linkages. The further addition of 10.0 mM GSH did not remarkably affect the DOX release profile, probably due to the prevalence of the pH effect over the GSH redox activity on the nanoparticle destabilization. To confirm these statements, kinetic parameters were investigated considering the DOX release from the nanoparticle structure as an equilibrium partition phenomenon between the carrier and the release media, and hypothesizing a first- or second-order kinetics (Equations (2) and (3)) [44]:

(2) $$\frac{M_{t}}{M_{0}}=F_{max}\left(1-e^{-\left(\frac{k_{R}}{F_{max}}\right)t}\right)$$

(3) $$\frac{M_{t}}{M_{0}}=\frac{F_{max}\left(e^{2\left(\frac{k_{R}}{\alpha }\right)t}-1\right)}{1-2F_{max}+e^{2\left(\frac{k_{R}}{\alpha }\right)t}}$$

*k_R_* was the release rate constant, *F_max_* corresponded to the maximum amount of released drug (*M_t_*/*M*_0_), while *α*, a measure of the physico–chemical affinity of the drug towards the nanoparticles and the solvent phase was calculated as follows (Equation (4)):

(4) $$\alpha =\frac{F_{max}}{1-F_{max}}$$

when *α* > 0 the drug release occurred.

As shown in Table 1, Equation (2) was more suitable to describe the experimental data (R^2^ > 0.97 in all conditions), indicating a predominant reversible first-order kinetics.

In detail, compared the condition of high nanoparticle stability (pH 7.4, *F_max_* = 0.33), greater *F_max_* values (>0.9) were recorded in all experimental conditions mimicking the intracellular microenvironment, confirming that the application of external stimuli (pH and/or GSH) resulted in the nanoparticle destabilization and in an enhanced drug release. In these conditions, the affinity for the release media were significantly higher (*α* from 13 to 31), while *α* value of 0.51 was recorded in physiological medium. Moreover, at pH 7.4 (either in the presence or absence of GSH), *k_R_* was reduced to half of the values recorded at pH 5.0, as a confirmation of the prevalence of the hydrolytic activity induced by the acidic pH over the GSH reductive effect.

### 2.4. Cell Viability Experiments and Apoptotic Assay

**Figure 6:**
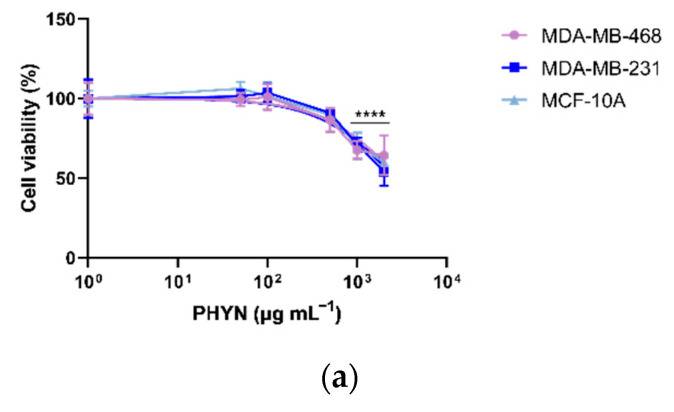

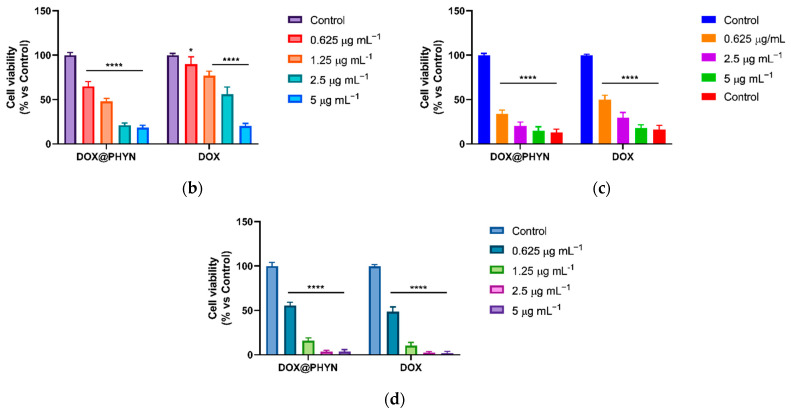
(**a**) Cell viability assessment of MDA-MB-231, MDA-MB-468 and MCF-10A exposed to PHYN (from 0.05 to 2 mg mL^−1^) for 72 h; cell viability assessment of (**b**) MDA-MB-231, (**c**) MDA-MB-468 and (**d**) MCF-10A exposed to DOX@PHYN or DOX (from 0.625 to 5 µg mL^−1^), as indicated, for 72 h. Cell viability was evaluated by MTT assay. Values represent means ± SD (n = 3) from three independent experiments. * *p* < 0.05; **** *p* < 0.0001 vs. control.

Based on the above-mentioned evidence concerning both the targeted drug delivery mediated by CD44 receptors and the pH/redox-responsive release, we further assessed the anticancer performance of the nanoparticles, by comparing the effect of free DOX, PHYN and DOX@PHYN on the viability of cancer and healthy cells (Figure 6).

The cells used display different features regarding the pH/redox state. Of note, MDA-MB-231 cells are marked by high glycolytic activity with consequently low pH compared to non-tumorigenic MCF-10A cells [45]; conversely, MDA-MB-468 cells are characterized by high GSH levels with respect to both MDA-MB-231 and MCF-10A [46].

First, the safety of the PHYN formulation was evaluated, finding no interference with cell viability at concentrations lower than 0.5 mg mL^−1^ (Figure 6a). Thus, the subsequent experiments with the DOX@PHYN were performed at nanoparticle concentrations below this value.

The inhibitory effect of DOX@PHYN on cell proliferation was compared to that of free DOX at the same concentration (Figure 6b–d), and the calculated half-maximal inhibitory concentration (IC_50_) values of the free and loaded drug are reported in Table 2.

**Figure 7:**
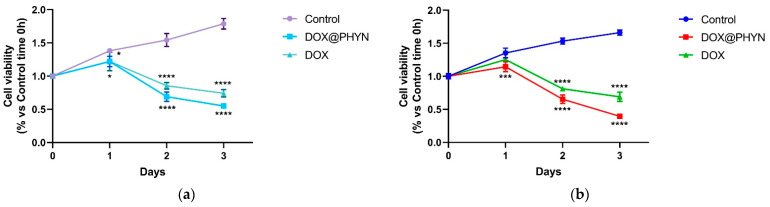
Cell viability of (**a**) MDA-MB-231 and (**b**) MDA-MB-468 exposed to DOX@PHYN or DOX at IC_50_. Cell viability was assessed by MTT assay. Values represent means ± SD (n = 3) from three independent experiments. * *p* < 0.05; *** *p* < 0.001; **** *p* < 0.0001 vs. control.

Our results show that the cytotoxic effect mediated by DOX@PHYN in MCF-10A, follows a comparable trend to that highlighted by free DOX (Figure 6d and Table 2), while a marked reduction in IC50 values was observed in both cancer cell lines, with values de-creasing almost two- (MDA-MB-468, Figure 6c) and three- fold (MDA-MB-231, Figure 6b), respectively. This effect is in good agreement with both the more efficient CD44-mediated internalization in cancer cells and the pH/redox-responsive release. Subsequently, in order to monitor the cytotoxic effects over time, cell viability was assessed over three days. After treatment with free DOX or DOX@PHYN at IC_50_ DOX equivalent concentrations, the growth curves of MDA-MB-231 and MDA-MB-468 cells showed an initial reduction in cell proliferation in the first 24 h (more marked in MDA-MB-468), followed by a cytotoxic effect (Figure 7).

The obtained results were consistent with the different biological features of the tested tumor cell lines. The highest levels of CD44 in MDA-MB-231 were responsible for stronger stemness power, as well as for higher cellular uptake and cytotoxic activity of DOX@PHYN. On the other hand, although lower CD44 levels were found in MDA-MB-468 cells, the marked mitochondrial metabolism and higher GSH levels required to maintain the optimal cellular redox state [46] resulted in an enhanced cytotoxic effect due to the higher release of the chemotherapeutic agent at the intracellular level.

**Figure 8:**
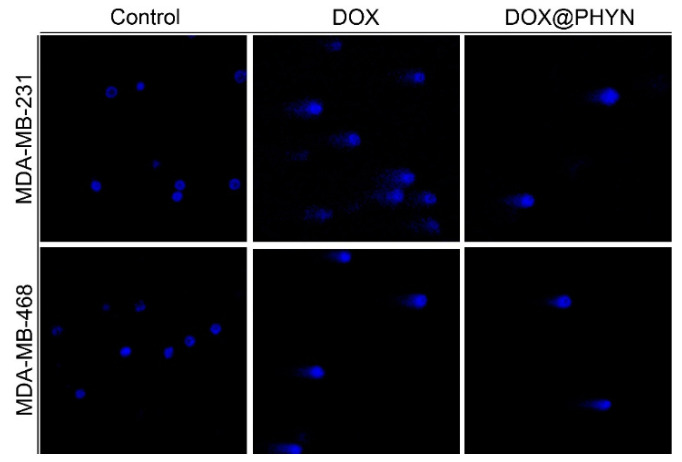
Triple negative breast cancer cells treated at IC_50_ values with DOX@PHYN or DOX for 3 days were subjected to the comet assay. DAPI was used to stain nuclei. Pictures were obtained at 20×. Scale bar 25 µm.

Finally, in order to assess whether the DOX@PHYN preparation was able to exert a cytotoxic effect by inducing apoptosis cell death, consistent with the typical effects medi-ated by free DOX [47], a comet assay was performed. This is a rapid and sensitive technique used for evaluating DNA damage in individual cells [48,49]. After 72 h of treatment with DOX@PHYN or free DOX, at their respective IC_50_ values, MDA-MB-231 and MDA-MB-468 cells showed the typical tail representing DNA fragmentation of the last event of the apoptotic process, in both treatments (Figure 8).

## 3. Materials and Methods

### 3.1. Synthesis of Oxidized Hyaluronic Acid

oxHA was prepared via oxidation reaction with sodium periodate (NaIO_4_) [50]. Briefly, 10.0 mL HA (MW 10 kDa, 0.2 g, 0.25 mmol) water solution was added with 3.0 mL NaIO_4_ (0.1 g, 0.25 mmol) water solution. The mixture was incubated for 24 h in the dark under magnetic stirring at room temperature and ethylene glycol was added and reacted for 1 h to stop the reaction. The resulting solution was purified by dialysis (MWCO 3.5 kDa, Medicell International LTD, London, UK) against water at 20 °C for 72 h, and finally freeze-dried (98% yield). ^1^H-NMR and FT-IR spectra were recorded on a Bruker Avance 300 (Bruker Italy, Milan, Italy) at 25 °C using D_2_O as solvent and a Jasco FT-IR 4200 (Jasco, Easton, MD, USA), respectively.

HA was purchased from Lifecore Biomedical (Chaska, MN, USA). All other chemicals were from Merck/Sigma–Aldrich (Darmstadt, Germany).

### 3.2. Preparation of Unloaded and Loaded Nanoparticles (PHYN and DOX@PHYN)

A modified thin film method was employed to prepare HA-PDC nanoparticles (PHYN). Briefly, 10.0 mg PDC were dissolved in 10.0 mL chloroform. The organic solvent was evaporated by rotary evaporation and the film was further dried overnight in vacuum. The film was hydrated using 5.0 mL oxHA solution (0.5 mg mL^−1^) in PBS (0.01 M, pH 7.4) by a probe sonicator at 200 W for 2 min in an ice bath. Then, an excess cystamine dihydrochloride (Cys, 1.5 mg) was added and incubated for 24 h under magnetic stirring at room temperature.

The obtained PHYN were purified by dialysis (MWCO 12–14 kDa, Medicell International LTD, London, UK) against phosphate buffer (0.01 M, pH 7.4) at room temperature.

For ^1^H-NMR analysis, the obtained PHYN nanoparticle solution was freeze-dried and the residue was reconstituted in a mixture of DMSO-d_6_ and D_2_O.

For the reduction of disulfide bonds within Cys moieties, 5.0 mg PHYN was added to a 5.0 mL 10 mM 2-mercaptoethanol solution in PBS (pH 7.4, 0.01 M) and incubated at 25 °C for 2 h [51]. Then, after exhaustive dialysis (MWCO 12–14 kDa, Medicell International LTD, London, UK) against PBS (pH 7.4, 0.01 M), Ellman’s assay was performed as follows [35]: 250 μL of dialyzed solution was mixed with 250 μL NaH_2_PO_4_/Na_2_HPO_4_ phosphate buffer (pH 8.0, 0.5 M) and 500 μL Ellman’s reagent consisting of 0.3 mg/mL DTNB in NaH_2_PO_4_/Na_2_HPO_4_ phosphate buffer (pH 8.0, 0.05 M). The reaction was allowed to stand for 2 h at 25 °C, filtered and the absorbance was measured at 420 nm on an Evolution 201 spectrophotometer (ThermoFisher Scientific, Hillsboro, OR, USA) operating with 1.0 cm quartz cells. The amount of thiol moieties was calculated from a calibration curve elaborated from solutions of cysteine (0.05 to 0.45 mM).

DOX-loaded nanoparticles (DOX@PHYN) were prepared following the same procedure, using 5.0 mL oxHA solution (0.5 mg mL^−1^) in PBS (0.01 M, pH 7.4) containing 0.2 mg mL^−1^ DOX hydrochloride (DOX) to hydrate the thin film. DOX encapsulation efficiencies (EE%) were determined employing the dialysis technique [52]. Briefly, 5.0 mL drug loaded PHYN were poured into a dialysis bag (MWCO 3.5 kDa, Medicell International LTD, London, UK) and immersed in 25.0 mL PBS (0.01 mM, pH 7.4) under magnetic stirring until no drug was recorded in the recipient medium (1 h). Then, 3.0 mL purified and not-purified nanoparticles were diluted with 25.0 mL methanol, in order to disassemble the nanoparticle structure, and the DOX amount was determined by fluorescence method.

The entrapment efficiency (*EE*%) was calculated according to the following Equation (5):

(5) $$EE\left(\%\right)=\frac{ND-D}{ND}\times 100$$

*ND* and *D* represent the drug concentrations before and after the dialysis, respectively.

Morphological and dimensional analyses of nanoparticles were performed by TEM and DLS, and surface charge determined by zeta potential measurement.

For morphological analysis, a drop of the nanoparticle dispersion was placed on a Cu TEM grid (200 mesh, Plano GmbH, Wetzlar, Germany), removing the exceeding sample by filter paper. A drop of phosphotungstic acid solution (2% *w*/*v*) was deposited on the carbon grid for 2 min, the sample was dried on air and the thin film was observed on HRTEM/Tecnai F30 (80 kV) (FEI company, Hillsboro, OR, USA).

The size distribution was determined using a 90 Plus Particle Size Analyzer DLS equipment (Brookhaven Instruments Corporation, Holtsville, NY, USA) at room temperature, operating with a 658 nm laser beam and measuring the autocorrelation function at 90°. The polydispersity index (PDI) was obtained by fitting the instrumental data by the inverse Laplace transformation and Contin methods. PDI values ≤ 0.3 indicate homogenous and mono-disperse populations [53].

The ζ-potential of the formulation was measured with the laser Doppler electrophoretic mobility measurements using the Zeta-Sizer ZS (Malvern Instruments Ltd., Malvern, UK), at 25.0 ± 0.1 °C. ζ-potential values were calculated by the instrument software, using the Helmholtz–Smoluchosky equation. All analyses were carried out in triplicate and expressed as mean ± standard deviation.

The colloidal stability was evaluated at 25 °C for a period of 30 days. Samples were withdrawn at definite time intervals (1, 7, 14 and 30 days) and the ζ-potential and the mean of hydrodynamic diameter of vesicles were determined as previously described.

All chemicals were purchased from Merck/Sigma–Aldrich (Darmstadt, Germany).

### 3.3. Cell Cultures

Triple negative breast cancer cell lines (MDA-MB-231 and MDA-MB-468) and human mammary epithelial cells (MCF-10A) were purchased from the American Culture Collection (ATCC, Manassas, VA, USA). MDA-MB-231 cells were cultured in DMEM/F12 supplemented with 10.0% fetal bovine serum (FBS), 2.0 mM l-glutamine, and 1.0% penicillin/streptomycin. MDA-MB-468 cells were cultured in DMEM high glucose supplemented with 10.0% FBS, 2 mM l-glutamine, 1.0% penicillin/streptomycin and 1.0% sodium-pyruvate. MCF-10A cells were cultured as previously reported [54].

All chemicals were purchased from Merck/Sigma–Aldrich (Darmstadt, Germany).

### 3.4. Immunoblotting Analysis

MDA-MB-231, MDA-MB-468 and MCF-10A cells were grown to 70–80% confluence, lysed and subsequently subjected to immunoblotting analysis as previously reported [55]. CD44 (Thermo Fisher Scientific, Waltham, MA, USA) specific antibody was used at 1:250 dilutions. Equal loading and transfer were confirmed by incubation with 1:1000 anti-β-actin antibody (Santa-Cruz Biotechnology, Santa Cruz, CA, USA). Antigen–antibody complexes were detected as previously described [56].

### 3.5. Confocal Microscopy Analysis

Triple negative breast cancer cells and MCF-10A cells were seeded upon coverslips inside 6-well plates with a density of 1.0 × 10^5^ cells/well and cultured overnight in complete medium. Then, cells were treated for 1 h with free DOX or DOX@PHYN at 0.5 µg/mL DOX equivalent concentration. At the end of treatment, cells were fixed for 15 min at 37 °C using 4.0% paraformaldehyde. Then, cells were washed in PBS and slides were visualized at 20× using a fluorescent microscope (DOX excitation at 485 and emission and 595 nm). A DAPI solution (0.2 µg/mL) was employed to stain the nuclei. To confirm CD44 receptor-mediated uptake, breast cells were pretreated with an excess HA for 1 h, in order to compete with the CD44 receptor as previously reported [57,58]. Subsequently, cells were treated with DOX@PHYN at 0.5 µg/mL DOX equivalent concentration, and then subjected to confocal microscopy analysis.

### 3.6. Release Experiments

In four different experiments, 2.0 mL DOX@PHYN dispersion was loaded in a dialysis bag (MWCO 3.5 kDa) and dialyzed against 10.0 mL phosphate (0.01 M, pH 7.4) or acetate (0.01 M, pH 5.0) buffer containing GSH at different concentrations (0 mM and 10.0 mM) at 37 °C in a beaker with constant stirring. At suitable time intervals, 0.5 mL release medium were withdrawn, replaced with fresh medium and the amount of released drug quantified by fluorescence spectrometer (λ_exc_ = 480 nm; λ_em_ = 590 nm) using a standard calibration curve of DOX (2–30 μM).

Experiments were performed under sink conditions.

### 3.7. Cell Viability and Growth Curves

Breast cancer and healthy cell lines were seeded in 48-well plates with a density of 2.0 × 10^4^ cells/well and cultured overnight in complete medium. Then, cells were treated with PHYN (from 0.05 to 2.0 mg mL^−1^), free DOX or DOX@PHYN from 0.625 to 5.0 µg mL^−1^ DOX equivalent concentration, for 72 h. Cells were then subjected to MTT assay, as previously described [59]. Sigmoidal dose–response curves used to calculate IC_50_ values for each cell line were obtained by non-linear regression analysis using GraphPad Prism 8 (GraphPad Inc., San Diego, CA, USA). Growth curve experiments were assessed by treating triple negative breast cancer cells with DOX@PHYN or DOX at IC_50_ values obtained from a cell viability assay for each cell line exposed to free DOX. Cell viability was assessed as described above, measuring viability over three days.

### 3.8. Comet Assay

3.0 × 10^5^ cells/well of MDA-MB-231 and MDA-MB-468 cell lines were treated for 72 h with DOX@PHYN or DOX at their respective IC_50_ values and then subjected, as previously described [60], to the comet assay, a technique widely used to assess DNA fragmentation [61,62]. Cells were visualized and pictures were obtained at 20× on a fluorescent microscope.

### 3.9. Statistical Analysis

All results were expressed as means ± SD (standard deviation), obtained over ≥three independent experiments, with ≥three replicates, unless otherwise stated. By using the one-way analysis of variance (ANOVA) test, differences among means were tested for statistical significance. *p* ≤ 0.05 was considered statistically significant.

## 4. Conclusions

In this work, a new CD44-targeted nanoparticle formulation was prepared combining a PDC bilayer structure with a cystamine-crosslinked oxHA, and proposed as a pH/redox-responsive delivery device of DOX HCl. The nanometric size of the obtained DOX@PHYN particles ensured their accumulation in tumor tissue by EPR effect, while their composition enabled the active targeting of DOX to cancer cells via both CD44 receptor interactions and the ability to respond to pH and GSH concentration. Cell viability experiments were performed on healthy and cancer cell lines, showing that the DOX encapsulation resulted in an almost unmodified IC_50_ value for MCF-10A, and in a significant improvement of the cytotoxic effect on MDA-MB-231 and MDA-MB-468. Cell uptake experiments confirmed the CD44-mediated internalization of nanoparticles in cancer cells, while the apoptotic mechanism of cell death, typical of doxorubicin treatment, was confirmed by comet assay. Although more in vivo investigations are required to confirm these interesting findings, the whole of the reported results clearly prove the suitability of the proposed nanoformulation as a tool to improve the therapeutic performances of conventional anticancer drugs.

## Figures and Tables

**Table 1:** R^2^ values and kinetic parameters for Equations (1) and (2).

Mathematical Model	Parameter	DOX			
		pH 7.4		pH 5.0	
		(GSH) 0 mM	(GSH) 10 mM	(GSH) 0 mM	(GSH) 10 mM
Equation (2)	R^2^	0.97518	0.98428	0.98652	0.98273
	*F_max_*	0.33989	0.96906	0.93295	0.96913
	*α*	0.51	31.32	13.91	31.39
	*k_R_* (10^−2^)	0.472	0.408	1.174	0.996
Equation (3)	R^2^	0.96441	0.38416	0.96811	0.95466

**Table 2:** Cytotoxic activity of DOX@PHYN and DOX.

Cell Line	IC_50_ (μg mL^−1^)	
	DOX	DOX@PHYN
MCF-10A	0.3377 0.2404 to 0.4529 ^(a)^	0.4310 ^(b)^ 0.3160 to 0.5688 ^(a)^
MDA-MB-231	2.808 2.214 to 3.584 ^(a)^	1.022 ^(c)^ 0.9022 to 1.156 ^(a)^
MDA-MB-468	0.5947 0.5158 to 0.6820 ^(a)^	0.3508 ^(c)^ 0.2972 to 0.4093 ^(a)^

^(a)^ 95% confidence intervals; ^(b)^ *p* > 0.05 vs. DOX IC_50_; ^(c)^ *p* < 0.05 vs. DOX IC_50_.

## Data Availability

Data are available from the authors.
